# The immunosuppressive cytokine interleukin-4 increases the clonogenic potential of prostate stem-like cells by activation of STAT6 signalling

**DOI:** 10.1038/oncsis.2017.23

**Published:** 2017-05-29

**Authors:** G Nappo, F Handle, F R Santer, R V McNeill, R I Seed, A T Collins, G Morrone, Z Culig, N J Maitland, H H H Erb

**Affiliations:** 1 The Cancer Research Unit, Department of Biology, University of York, York, UK; 2 Department of Experimental and Clinical Medicine, Laboratory of Molecular Haematopoiesis and Stem Cell Biology, Magna Graecia University, Catanzaro, Italy; 3 Division of Experimental Urology, Department of Urology, Medical University of Innsbruck, Innsbruck, Austria; 4 Jack Birch Unit for Molecular Carcinogenesis, Department of Biology, University of York, York, UK; 5 Hull York Medical School, University of Hull, Hull, UK; 6 Department of Urology and Pediatric Urology, University Medical Center Mainz, Mainz, Germany

## Abstract

Interleukin-4 plays a critical role in the regulation of immune responses and has been detected at high levels in the tumour microenvironment of cancer patients, where concentrations correlate with the grade of malignancy. In prostate cancer, interleukin-4 has been associated with activation of the androgen receptor, increased proliferation and activation of survival pathways such as Akt and NF-κB. However, its role in therapy resistance has not yet been determined. Here we investigate the influence of interleukin-4 on primary epithelial cells from prostate cancer patients. Our data demonstrate an increase in the clonogenic potential of these cells when cultured in the presence of interleukin-4. In addition, a Phospho-Kinase Array revealed that in contrast to previously published work, signal transducer and activator of transcription6 (STAT6) is the only signalling molecule activated after interleukin-4 treatment. Using the STAT6-specific inhibitor AS1517499 we could confirm the role of STAT6 in increasing colony-forming frequency. However, clonogenic recovery assays revealed that interleukin-4 does not rescue the effects of either irradiation or docetaxel treatment. We therefore propose that although the interleukin-4/STAT6 axis does not appear to be involved in therapy resistance, it does play a crucial role in the colony-forming abilities of the basal cell population in prostate cancer. IL-4 may therefore contribute to disease relapse by providing a niche that is favourable for the clonogenic growth of prostate cancer stem cells.

## Introduction

Prostate cancer (PCa) is one of the most frequent malignancies in males.^1^ Treatment is strongly dependent on tumour stage, patient age, overall patient health and tumour risk assessment.^2, 3, 4^ The most commonly used treatment options for PCa are radical prostatectomy, radiation therapy, multiple endocrine therapies and chemotherapy with docetaxel. Although most PCa patients respond initially to androgen deprivation treatment, the cancer inevitably recurs and progresses to highly aggressive castration-resistant PCa, for which only palliative therapeutic options exist.^3, 5^ However, the exact mechanism behind the development of castration-resistant PCa is still unclear. One possible reason for this progression is that currently used therapies have only been designed to target androgen receptor-positive luminal cells in the cancer.^5^ However, several studies have demonstrated that a small population of primitive cells with a basal phenotype (characterized by AR^−^, CD49f^+^, CD44^+^, CKs 5/14^+^ and p63^+^ markers) exist within the tumour, and have the capacity to evade current therapies.^6, 7, 8^ These rare cells (<1%) have been shown to possess a higher regenerative potential and express tumour markers (including AMACR and theTMPRSS2-ERG fusion gene).^9, 10, 11^ Similar to benign prostate tissue, the basal cells (CD44^+^/CD49f^+^) from malignant areas can further be subgrouped by high expression of α2β1 integrin complex (CD49b), which results in a rapid adhesion to collagen.^12^ The basal compartment can also be further fractionated into stem cells (SC, CD49b^high^/CD133^+^), the highly proliferative transit-amplifying cells (TA, CD49b^high^/CD133^−^) and committed basal cells (CB, CD49b^low^/CD133^−^).^5, 10^ CB cells have also been reported in several studies as intermediate cells, and harbour luminal and basal markers, such as cytokeratins 5, 14 and 18.^5, 10^ Interestingly, SC isolated from malignant areas (cancer stem cells, CSC) are highly invasive, have a shorter population doubling time and a distinct messenger RNA (mRNA) and microRNA profile compared to normal SC, and in addition can form tumours in mice.^10, 11, 13^

Recent clinical studies have demonstrated that inflammation is not only linked to the development of cancer, but is also an indicator of poor prognosis.^8, 14^ Chronic inflammation has been associated with the production of a variety of cytokines by inflammatory cells, including interleukin (IL)-1, IL-6 and IL-4.^15^ In addition to the action on immune cells, cytokines modulate the different cells types within the tumour microenvironment, and are able to induce cell transformation.^15^ For example, increased IL-6 levels have been observed in PCa tissues, and are suggested to influence growth and survival pathways.^16^ IL-6 expression levels in prostate tissue also (i) correlate with Gleason score and biochemical recurrence, (ii) influence tumour initiation and (iii) affect clonogenic recovery after docetaxel treatment of PCa stem-like cells.^16, 17, 18^

IL-4 is a multifunctional cytokine that plays a critical role in the regulation of immune responses.^19^ Cytokine binding to the IL4R alpha-chain (IL4Rα) subunit results in the activation of mediators of cell growth, resistance to apoptosis, gene activation and differentiation.^19^ The activated signal pathways include AKT, p44/42 MAPK, NF-κB and the JAK/STAT6 pathways, which represents the main mediator of IL-4 signalling in immune cells.^19^ Elevated levels of IL-4 (normally produced by tumour-infiltrating lymphocytes) have been documented in patients with progressive PCa,^20, 21, 22^ and *in vitro* studies using PCa cell lines have demonstrated that IL-4 activates NF-κB and androgen receptor in a ligand-independent manner.^23^ Treatment of androgen-sensitive LNCaP cells with IL-4 increased the expression of the co-activators CBP/p300 and their histone acetyltransferase activity.^15, 24^ Overexpression of IL-4 resulted in increased proliferation of LNCaP and 22Rv1 cell lines,^25^ while IL-4 treatment also induced the proliferation of the androgen receptor-negative PC3 cells under nutrient-depletion stress.^26^

In this study, we investigated the physiological and molecular effects of the pleiotropic cytokine IL-4 on primary basal prostate cells, isolated from benign and malignant prostate biopsies. By simulating an IL-4-containing microenvironment (using IL-4 secreting feeder cells), we determined the effect of IL-4 on primary basal cell fate, cell mortality, cell invasion, and whether IL-4 signalling is involved in therapy resistance.

## Results

### IL4Rα expression in primary prostate epithelial cells

**Figure 1:**
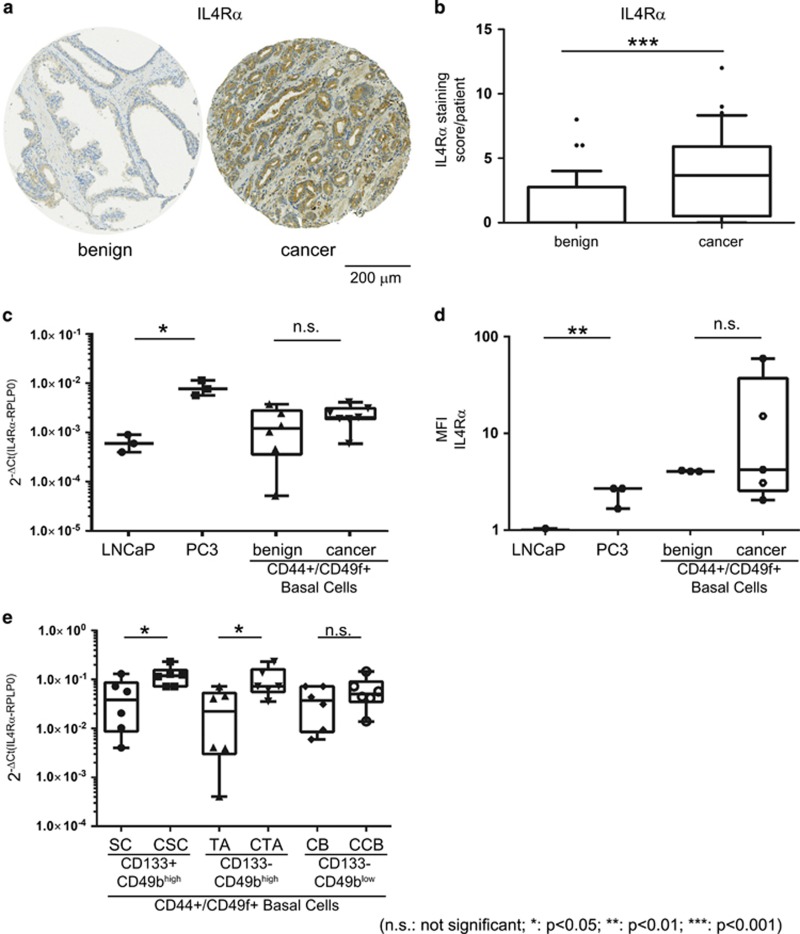
IL4Rα expression in prostate epithelial cells. (**a**) Immunohistochemical staining for IL4Rα of representative malignant tissue and adjacent benign tissue cores from a PCa patient. Scale bar=200 μm. (**b**) Evaluation of a TMA of malignant tissue and adjacent benign tissue cores from PCa patients (*n*=36) stained for expression of IL4Rα. Data are shown as box and whisker diagrams (10–90%, ***P<0.001, outliers are shown as dots). (**c**) IL4Rα mRNA levels in LNCaP (*n*=3), PC3 (*n*=3), benign (*n*=8) and malignant (*n*=8) primary prostate cell cultures normalised to RPLP0 expression. Data are shown as box and whisker diagrams (min to max; *P<0.05). (**d**) IL4Rα protein levels of LNCaP (*n*=3), PC3 (*n*=3), benign (*n*=4) and malignant (*n*=5) primary prostate cell cultures analysed by flow cytometry. Data are shown as box and whisker diagrams (min to max; *P<0.05; **P<0.01; ***P<0.001). (**e**) IL4Rα mRNA levels of individual cell subpopulations from benign and malignant primary prostate cell cultures (*n*=6). Data are shown as Box and whisker diagrams (min to max; *P<0.05). CCB, cancer committed basal; CTA, cancer transit amplifying; NS, not significant.

IL-4 acts by binding to a heterodimeric receptor complex, composed of the IL4Rα and the common gamma chain shared by several IL receptors.^27^ To evaluate IL4Rα expression patterns in benign and malignant prostate tissue, we investigated expression of IL4Rα in a tissue microarray (TMA) containing both malignant and adjacent benign areas from 36 PCa patients (Figure 1a). The TMA showed that IL4Rα is mainly expressed in luminal cells and significantly elevated in cancerous compared to adjacent benign areas (Figure 1b). The bulk of prostate adenocarcinomas consist of luminal cancer cells, precluding analysis of the IL4Rα expression levels in rare cell populations such as the basal cell compartment in malignant areas.^12, 28^ Due to these technical limitations, the detection of rare basal cell populations in human tissue by immunohistochemical methods is highly challenging. To overcome these limitations, we made use of primary cell cultures from benign prostate hyperplasia and PCa tissue specimens to amplify the number of basal epithelial cells. In contrast to the findings from the TMA (Figures 1a and b) IL4Rα mRNA (Figure 1c) and protein levels (Figure 1d) were not significantly different between benign and cancerous CD44^+/^CD49f^+^ basal cells, which both showed comparable expression levels to PC3 cells. Surprisingly, in view of the TMA data, LNCaP cells expressed only minimal levels of IL4Rα (Figures 1c and d). To investigate IL4Rα expression in the individual cell subpopulations, we separated SC, TA and CB according to their surface markers, as previously described.^12, 27, 28^ The functional isolation of SC, TA and CB subpopulations (based on their clonogenic potential) was confirmed (Supplementary Figure 1A), and quantitative real-time PCR analysis of the SC, TA and CB subpopulations revealed that receptor expression was significantly increased in the cancer (C)SC and cancer transit amplifying subpopulation within the basal cell population compared with their benign counterparts (Figure 1e). We were unable to stratify the clonogenicity of the basal populations or IL4Rα expression using the expression of another common prostate (basal) stem cell marker CD49f,^8^ which marked all basal cells under these serum-free culture conditions (Supplementary Figure 1B).^8^

### IL-4 increases the clonogenic potential of primary PCa cells

**Figure 2:**
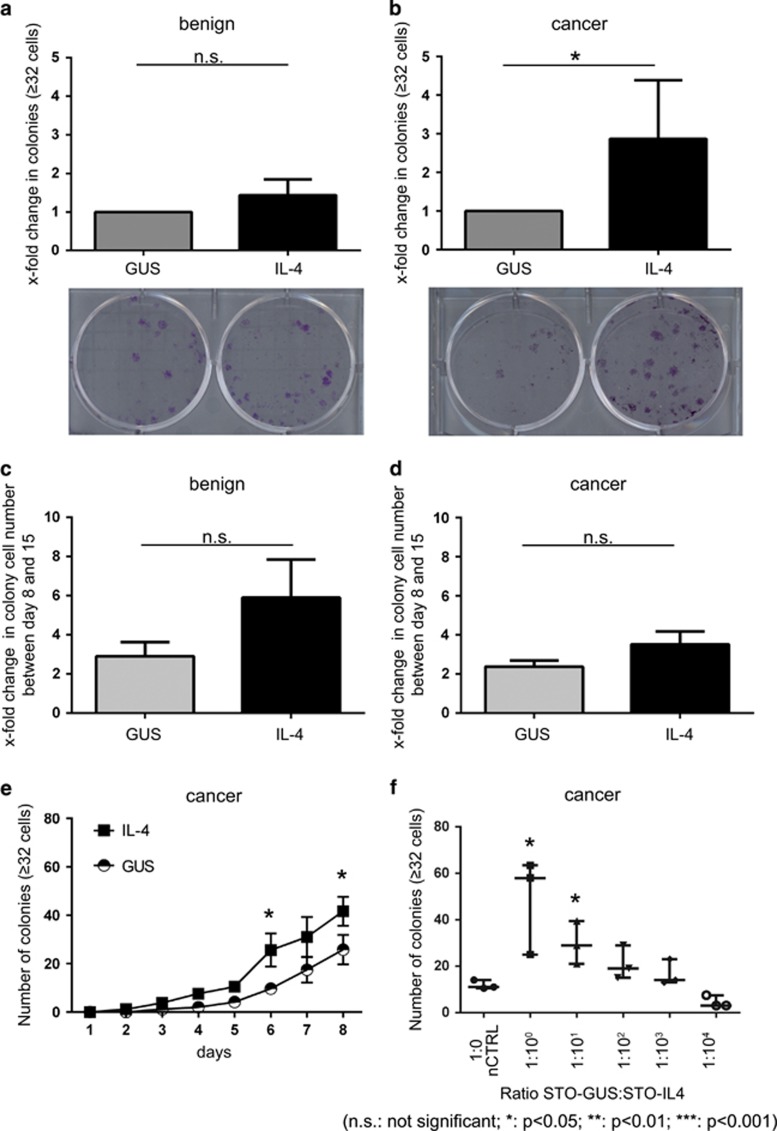
IL-4 increases clonogenic potential of primary PCa cells. (**a**) Clonogenic assays of benign primary prostate cells (*n*=4) co-cultured with STO-GUS or STO-IL-4 feeder cells. The number of colonies (⩾32 cells) was scored 15 days after plating. The results are expressed as fold change in the number of colonies and are normalised to the STO-GUS control. A representative crystal violet staining is shown at day 15. Data are shown as mean±s.d. (*P<0.05). (**b**) Clonogenic assays of malignant primary prostate cells (*n*=4) co-cultured with STO-GUS or STO-IL-4 feeder cells. The number of colonies (>32 cells) was scored 15 days after plating. The results are expressed as fold change in the number of colonies and are normalised to the STO-GUS control. A representative crystal violet staining is shown at day 15. Data are shown as mean±s.d. (*P<0.05). (**c**) Average colony growth of benign primary prostate cells (*n*=3) co-cultured with STO-GUS or STO-IL-4 feeder cells. Cell numbers from two colonies from each condition were counted at day 8 and 15, and the increase in cell number of one colony expressed as fold change. Data are shown as mean±s.d. (**d**) Average colony growth of malignant primary prostate cells (*n*=3) co-cultured with STO-GUS or STO-IL-4 feeder cells. Cell numbers from two colonies from each well were counted at day 8 and 15 and the colony growth expressed as fold change. Data are shown as mean±s.d. (**e**) Time course of colony formation (⩾32 cells) in primary PCa cell cultures (*n*=3) co-cultured with STO-IL-4 or STO-GUS for 8 days. The results are expressed as total number of colonies. Data are shown as mean±s.e.m. (*P<0.05). (**f**) Clonogenic assay of primary PCa cells (*n*=3) co-cultured with a combination of STO-GUS and STO-IL-4 feeder cells in different ratios (1:1–1:10 000). The number of colonies (⩾32 cells) was scored 15 days after plating. The results are expressed as total number of colonies. Data are shown as mean±s.e.m. (*P<0.05). NS, not significant.

To simulate an IL-4-containing microenvironment, a human IL-4 complimentary DNA construct was inserted into murine STO cells by lentiviral transduction. IL-4 expression and bioavailability as a secreted protein were confirmed by quantitative real-time PCR and ELISA (Supplementary Figures 2A and B). The newly generated STO cell lines, STO-GUS (control cell line) and STO-IL-4 (IL-4 secreting cell line) were used as feeder layers for the primary prostate epithelial cultures in subsequent experiments in order to investigate the influence of IL-4 on clonogenic potential. After 15 days of culture, benign primary cells co-cultured with STO-IL-4 cells showed a minor increase in colony forming efficiency (CFE), compared to cells co-cultured with STO-GUS cells (Figure 2a; Supplementary Figure 3A). In contrast, primary PCa cells showed a significantly (~3-fold) higher clonogenic potential in response to STO-IL-4 (Figure 2b; Supplementary Figure 3B). Similar effects were seen when PCa cells were co-cultured with untransduced STO cells in the presence of 5 ng/ml recombinant IL-4 (Supplementary Figure 3C). To investigate whether the presence of IL-4 affects the proliferative ability of primary cells, colony growth was tracked over time. Surprisingly, there was no significant difference between the size of colonies in STO-GUS or STO-IL-4 co-cultured cells, or between cancer and benign cells (Figures 2c and d). Similar results were observed in cell lines and primary cells treated with 5 ng/ml exogenous IL-4 for 7 days (Supplementary Figures 3D**–**H). Moreover, [^3^H] thymidine incorporation assays (Supplementary Figures 3I–L) and MTT assays (Supplementary Figures 3M–P) of commonly used PCa cell lines treated with a concentration range of IL-4 confirmed that IL-4 is not able to affect cellular proliferation rate or viability, respectively. To further corroborate whether IL-4 expedites colony formation, the development of colonies was scored over time. After 6 days, there was a significant increase in colony number in primary PCa cells co-cultured with STO-IL-4 compared to controls (STO-GUS) (Figure 2e). Thus, IL-4 is able to increase CFE of primary PCa cells but once colonies have formed, IL-4 is unable to regulate the expansion of these colonies.

To investigate whether CFE of PCa cells is dependent on the concentration of IL-4, primary cancer cells were co-cultured with STO-IL-4/STO-GUS cells at different ratios (Figure 2f). For these experiments STO-IL-4 cells were diluted with STO-GUS cells to a maximum ratio of 1:10 000. The IL-4 concentrations of the supernatants were determined after 2 days by ELISA and showed a concentration range from 0.004  to 32 ng/ml (Supplementary Figure 4A). The results demonstrated that the number of colonies correlated (Supplementary Figures 4B and C; *R*^2^=0.867; *P*=0.021) with the concentration of IL-4 in the supernatant suggesting that the clonogenic potential of primary PCa cells is increased in a dose-dependent manner by IL-4. As IL-4 effects on benign cells were absent, subsequent experiments were performed with malignant cells only.

### IL-4 does not influence the migration or invasive potential of primary PCa cells

**Figure 3:**
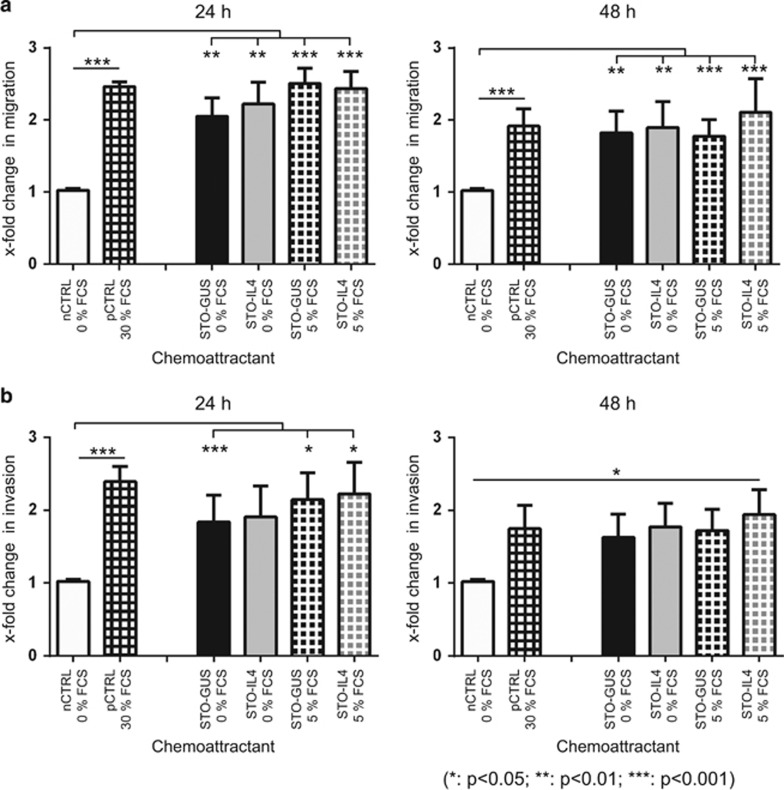
IL-4 does not influence the migration or invasion potential of primary PCa cells. (**a**) Fold change in migration of primary cancer cells after 24  and 48 h incubation compared to controls (*n*=4). Keratinocyte-serum free medium (KSFM) without added supplements served as a negative control for migration (nCTRL 0% FCS). KSFM supplemented with 30% FCS served as positive control (pCTRL 30% FCS; *P<0.05; **P<0.01; ***P<0.001). (**b**) Fold change in invasion through matrigel of primary PCa cells after 24  and 48 h incubation compared to controls (*n*=4). KSFM without added supplements served as a negative control for invasion (nCTRL 0% FCS). KSFM supplemented with 30% FCS served as positive control (pCTRL 30% FCS; *P<0.05; **P<0.01; ***P<0.001). nCTRL, negative Control, pCTRL, positive Control.

In order to determine whether IL-4 influences the chemotaxis of primary PCa cells, migration (Figure 3a) and invasion assays (Figure 3b) were performed with Boyden chambers in the presence of STO-IL-4 and STO-GUS acting as chemoattractants. Media with no supplements/chemoattractants were used as a negative control and medium supplemented with 30% fetal calf serum (FCS) was used as a positive control.^13^ STO cells in the absence of FCS had a similar effect on migration and invasion as 30% FCS. However, we were also unable to see any statistically significant effects on migration and invasion of primary PCa cells when STO-IL-4 was used as a chemoattractant. Noteworthy, this type of assay does not discriminate between the different subpopulations of primary PCa cells. We can therefore not exclude that SC niches do not need to secrete IL-4 in order to attract SC or CSC.

### IL-4 signalling does not affect the sensitivity of primary PCa cells to irradiation or docetaxel

**Figure 4:**
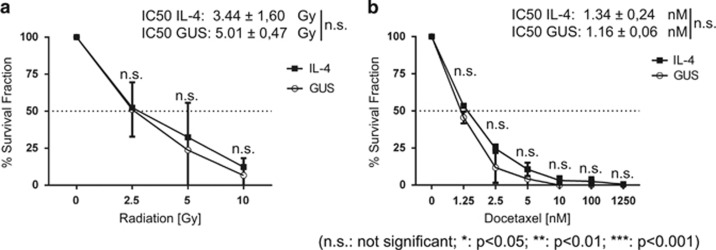
IL-4 does not influence the recovery potential of primary PCa after irradiation or docetaxel treatment. (**a**) Clonogenic recovery assay of primary PCa cells (*n*=3) co-cultured with STO-GUS or STO-IL-4 feeder cells after irradiation with 0, 2.5, 5 and 10 Gy. The number of colonies (⩾32 cells) was scored 10 days after plating. The results are expressed as % survival decrease in the number of colonies and normalised to no radiation (0 Gy) control. IC50 was calculated with GraphPad Prism from these results. (**b**) Clonogenic recovery assay of primary PCa cells (*n*=3) co-cultured with STO-GUS or STO-IL-4 feeder cells after 48 h treatment with different doses of docetaxel (0, 1.25, 2.5, 5, 10, 100 and 1250 nm). The number of colonies (>32 cells) was scored 10 days after plating. The results are expressed as % survival decrease in the number of colonies and normalised to no treatment (0 nm) control (*n*=3). IC50 was calculated with GraphPad Prism from these results. IC, inhibitory concentration; NS, not significant.

Multiple cytokines, such as IL-6, IL-8 and IL-4, have been shown to be expressed at higher levels after cancer treatment (for example radiation and chemotherapy (docetaxel)), and may play a crucial role in resistance mechanisms.^15, 20, 29^ In order to verify whether IL-4 may influence the recovery potential of primary cancer cells after irradiation, clonogenic recovery assays were performed after cells were exposed to increasing doses of γ-irradiation (2.5, 5, and 10 Gy) in the presence or absence of IL-4. As expected, γ-irradiation caused a significant decrease in colony numbers (expressed as relative survival fraction) (Figure 4a). However, there was no significant difference in clonogenic recovery between PCa cells co-cultured with STO-IL-4 (inhibitory concentration (IC)50 3.44±1.60 Gy) and the STO-GUS control (IC50 5.01±0.47 Gy).

To further corroborate whether IL-4 is able to affect the clonogenic recovery rate in response to a treatment, we repeated the assays after exposure to docetaxel, a standard of care chemotherapeutic intervention for castration-resistant PCa. A significant change in primary cell viability after 72 h treatment with increasing doses of docetaxel was observed (Supplementary Figure 5A). However, morphological observations (increase in nuclear size) and cell cycle analysis (G_2_ arrest) revealed that docetaxel was already effective at the lowest concentration (1.25 nm) tested after 24 h treatment (Supplementary Figures 5B**–**E). Subsequently, to investigate whether IL-4 may be involved in relapse after docetaxel treatment, clonogenic recovery assays were performed following an initial 48 h treatment with a concentration range from 1.25 nm to 1.25 μm docetaxel. As expected, a significant decrease in the number of colonies was observed after docetaxel treatment (Figure 4b). Similar to irradiation, no significant changes in either recovery potential or the IC50 between docetaxel-treated cells co-cultured with STO-IL-4 (IC50 1.34±0.24 nm) and co-cultured with STO-GUS (IC50 1.16±0.06 nm) could be observed (Figure 4b). We concluded that IL-4 signalling is not able to decrease the sensitivity of primary PCa cells in response to irradiation or docetaxel treatment.

### IL-4 treatment induces phosphorylation of STAT6 in PCa cells

**Figure 5:**
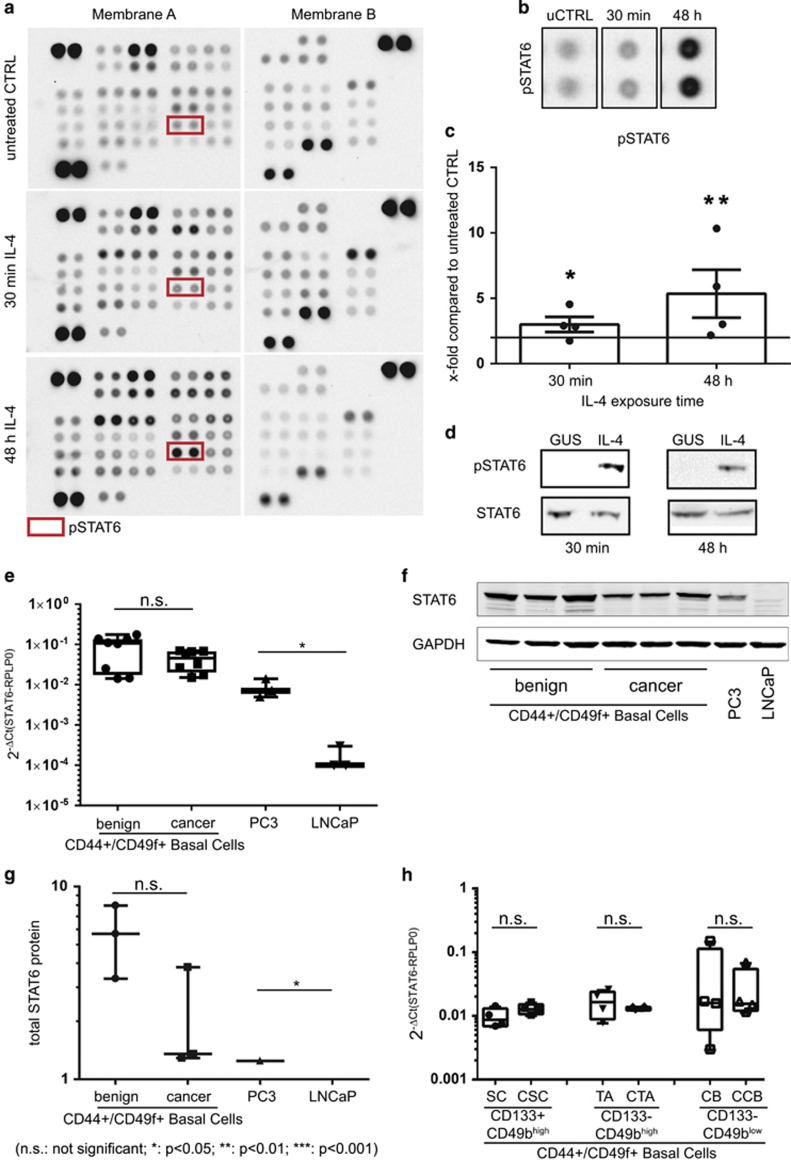
IL-4 treatment induces phosphorylation of STAT6 in Pca. (**a**) Representative membranes from the Human Phospho-Kinase Antibody Array. Samples were treated with 48 h-conditioned media from either STO-GUS controls (first lane) or STO-IL-4, for either 30 min (second lane) or 48 h (third lane). (**b**) Highlighted pSTAT6 dots on the representative membranes from the Human Phospho-Kinase Antibody Array. (**c**) Fold change of pSTAT6 after 30 min and 48 h of IL-4 exposure in the Human Phospho-Kinase Antibody Array compared to the untreated control (*n*=4 PCa). Data are shown as mean±s.d. (*P<0.05 **P<0.01). A two-fold change was chosen as the threshold for upregulation (black line). (**d**) Validation for pSTAT6 by Western blot analysis of primary PCa cells treated with conditioned media from STO-GUS or STO-IL-4 for 30 min or 48 h (*n*=1). (**e**) mRNA levels of STAT6 in LNCaP (*n*=3) and PC3 (*n*=3) cell lines, and benign (*n*=8) and malignant (*n*=8) primary prostate cell cultures. Data are shown as Box and whisker diagrams (min to max; *P<0.05). (**f**) Representative Western blot for STAT6 from primary prostate benign cells (*n*=3), primary PCa cells (*n*=3), PC3 (*n*=1) and LNCaP (*n*=1) cell lines. PC3 cells were used as a positive control and LNCaP cells as a negative control. (**g**) Protein levels of STAT6 analysed in primary prostate benign (*n*=3) and cancer cells (*n*=3) from the representative Western blot (Figure 5f). PC3 cells were used as a positive control and LNCaP cells as a negative control. Data are shown as Box and whisker diagrams (min to max; * *P*<0.05). (**h**) mRNA levels of STAT6 in individual cell subpopulations from benign and malignant prostate biopsies (*n*=4). Data are shown as Box and whisker diagrams (min to max). CCB, cancer committed basal; CTA, cancer transit amplifying; NS, not significant; Red rectangle, STAT6.

Next, we assessed which downstream signalling pathways are activated by IL-4 and may be involved in the increase in clonogenic potential observed in primary PCa cells. To this end, a Proteome Profiler Human Phospho-Kinase Array was utilised. Four primary PCa cell cultures from different patients were treated for either 30 min or 48 h with 48 h-conditioned media from STO-IL-4 cells. The array revealed that IL-4 treatment triggered a highly heterogeneous response in primary PCa cells, with only phosphorylation of signal transducer and activator of transcription6 (STAT6) identified as being significantly upregulated (*P*<0.05) in all samples and at all time points (Table 1; Figures 5a–c). Activation of STAT6 was validated by Western blot after treatment with IL-4-containing supernatant from STO-IL-4 for either 30 min or 48 h (Figure 5d).

Previous immunohistological studies have reported that STAT6 is overexpressed in PCa,^30^ and assessment of our TMA for STAT6 expression supported these results (Supplementary Figure 6). However, the data also showed a slight, statistically not significant decrease of both STAT6 mRNA (Figure 5e) and protein (Figure 5f) levels in cancer-derived primary cells compared to benign cells. Cancerous primary cells showed similar STAT6 protein expression to PC3 cells, despite the malignant primary cells demonstrating higher transcript expression. In contrast, LNCaP cells showed very-low STAT6 mRNA expression and no detectable protein (Figures 5e–g). In addition, no differences in STAT6 mRNA levels were observed between the fractionated subpopulations from cancer or benign cultures (Figure 5h). We concluded that STAT6 is highly expressed in primary PCa cells, albeit at lower levels than in benign cells, and can readily be activated by IL-4.

### Inhibition of phosphoSTAT6 reverses the IL-4-mediated increase in clonogenic potential

**Figure 6:**
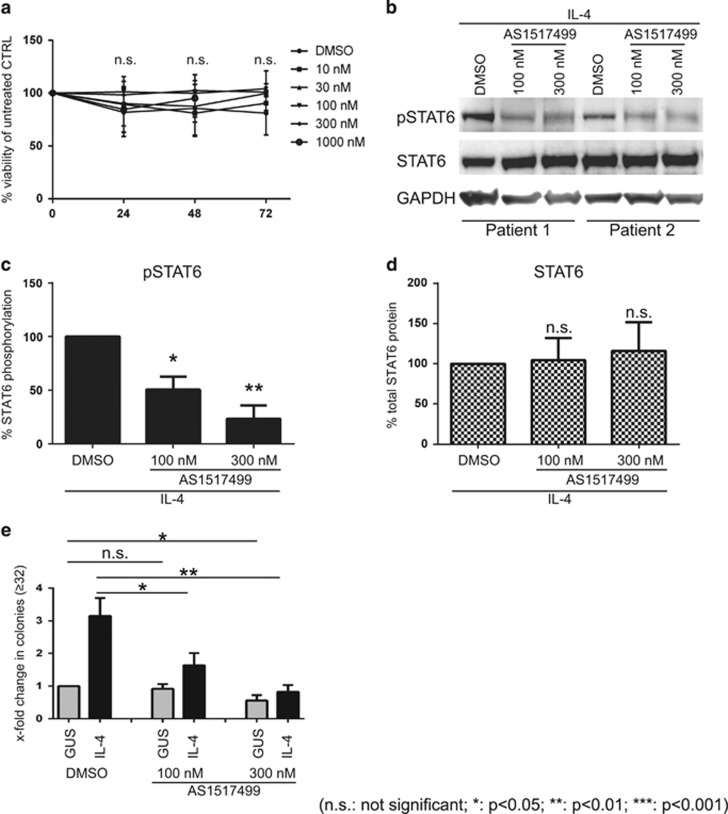
Inhibition of phosphoSTAT6 reverses the IL-mediated effects on clonogenic potential. (**a**) Cell viability of primary PCa cells treated with different doses of AS1517499 for 24, 48 and 72 h using the Alamar Blue Assay (*n*=3). (**b**) Two representative Western blots of phosphorylated and total STAT6 in primary PCa cells exposed to two different (100, 300 nm) doses of AS1517499 combined with STO-IL-4 supernatant for 30 min. (**c**) Densitometry analysis of pSTAT6 expression in AS1517499/IL-4-treated primary PCa cells compared to untreated controls (*n*=3; *P<0.05 **P<0.01). (**d**) Densitometry analysis of total STAT6 protein expression in AS1517499/IL-4 treated primary PCa cells compared to untreated controls (*n*=3). (**e**) Fold change in the number of colonies in primary benign and cancer prostate cells after 15 days of culture with two doses of AS1517499 (*n*=3 benign, *n*=3 cancer). (*P<0.05; **P<0.01; ***P<0.001). NS, not significant.

In order to investigate whether direct inhibition of STAT6 signalling can antagonize the effects of IL-4 on the clonogenic potential of primary PCa cells, a selective STAT6 inhibitor (AS1517499) was used at varying concentrations.^31^ After toxicity testing in primary cells (Figure 6a), two concentrations of the inhibitor (100  and 300 nm) were selected for further use, and their efficacy was verified by Western blot analysis for STAT6 phosphorylation following IL-4 exposure. While the inhibitor had no observable effect on cell viability (Figure 6a) and total STAT6 protein expression (Figures 6b and d), the levels of phosphorylated STAT6 decreased to 60 and 20% of initial expression levels following treatment with 100 and 300 nm AS1517499 respectively (Figures 6b and c).

As shown in Figure 2b, IL-4 increased the clonogenic potential of primary cancer cells. When PCa cells were co-cultured with STO-IL-4 in the presence of 100 nm of AS1517499, this increase in clonogenic potential was found to be significantly inhibited (*P*<0.05) (Figure 6e). Furthermore, the higher dose of AS1517499 (300 nm) showed an almost complete reversion of IL-4-increased clonogenic potential, to levels comparable to control (STO-GUS). Although 300 nm AS1517499 had a minor effect on clonogenic potential of the STO-GUS-co-cultured PCa cells, we concluded that the increase of the clonogenic potential of primary PCa cells in response to IL-4 is indeed mediated by STAT6 activation.

## Discussion

The role of important pro-inflammatory cytokines such as IL-1 and IL-6 in carcinogenesis has been extensively investigated.^15, 32^ In contrast, less is known about the effects of anti-inflammatory cytokines such as IL-4 on PCa cells. IL-4 is a pleiotropic cytokine produced by a subset of CD4^+^ T cells in response to receptor-mediated activation events. It induces a variety of responses in hematopoietic tissues, signalling mainly through the IL4Rα subunit, which is present in many tissue types including hematopoietic, endothelial, epithelial, muscle, fibroblast, hepatocyte and brain.^33, 34, 35^ Using a TMA, we were able to demonstrate that IL4Rα is significantly overexpressed in luminal PCa cells compared to cells from benign areas of the human prostate. Similar results have been observed in PCa xenograft models where IL4Rα is overexpressed in both androgen-dependent and -independent tumours.^36^ These results added PCa to the group of solid human tumours where IL4Rα is highly expressed, similar to breast, ovarian and colon cancers.^37, 38^ Interestingly, we found no significant difference in IL4Rα expression between benign and malignant primary basal prostate cells. However, fractionation of these rare cell populations revealed that stem-like cells isolated from cancers have significantly higher expression levels of IL4Rα, suggesting a potential tumourigenic role for IL-4/IL4Rα in PCa CSCs. Previously, IL-4/IL4Rα signalling activation was shown to promote clonogenic potential and metastatic colonisation in stem-like cells from human colon and mammary cancers.^38, 39, 40^ Similar to these findings, we showed that IL-4 significantly increased the clonogenic potential of PCa primary cells.

Former studies have demonstrated that IL-4 can induce NF-kB activation through activation of PI3K/AKT in PCa cell lines.^23^ In contrast, we found that IL-4 treatment induced only STAT6 phosphorylation in all the patient-derived cell cultures analysed (despite high heterogeneity between samples). This is supported by the findings of Ni *et al.*,^41^ who also showed significant levels of activated STAT6 in primary prostate tissues.

Here we describe a new role for IL-4-activated STAT6 in the regulation of clonogenic potential of primary PCa cells. Previous studies from our laboratory have revealed that only the progenitor subpopulations within primary benign and malignant cell cultures hold the ability to form colonies from single cells.^12, 13, 42^ This observation, combined with our results, leads to the hypothesis that IL-4 directly influences the CSC population by providing a micro-environmental stimulus to exit quiescence and initiate the formation of new colonies from a single cell. This adaptation to the IL-4-producing tumour microenvironment may play a critical role in the promotion of novel tumour-initiating foci, and possibly tumour initiation at distant sites. This hypothesis is supported by previous findings, which have demonstrated that (i) knockdown of STAT6 expression can inhibit tumour metastasis in PCa, (ii) STAT6^−^/^−^ mice are resistant to metastatic disease and (iii) STAT6 phosphorylation promotes metastatic potential in colon cancer cells.^43, 44, 45^ However, epithelial to mesenchymal transition has also been linked to increased clonogenicity, and may play a role in the observed results.^46^

*In vitro* and *in vivo* studies with the STAT6 inhibitor Leflunomide have reported reduced growth and promotion of apoptosis in PCa.^47^ However, Leflunomide is known to have non-specific effects and can alter targets other than STAT6.^30, 48, 49^ Recently, Nagashima *et al.*^49^ synthesized the potent and selective STAT6 inhibitor AS1517499, which has been utilised in this study. However, AS1517499 did not reduce proliferation as has been previously reported for Leflunomide. Despite this, we showed that AS1517499 was able to prevent the IL-4-activated STAT6-mediated increase of the clonogenic potential of primary PCa cells. Moreover, at a dose of 300 nm, AS1517499 decreased the clonogenic potential of primary basal PCa cells. Even though dose-dependent non-specific effects of AS1517499 cannot be excluded, it is more likely that the reduced clonogenic potential observed is due to the inhibition of basal STAT6 activation, which has been reported previously.^41^ These results support the hypothetical novel role of STAT6 in the regulation of the clonogenic potential of PCa.

Several markers have been identified in order to isolate the highly tumourigenic CSC from PCa cell lines, as well as primary PCa cells.^50^ However, these studies differ in both detection methods and the cell culture conditions for the CSC population, which has been shown to be highly adaptive to the microenvironment.^51^ The model used here was first introduced by Collins and colleagues in 1998, and the laboratory of Maitland and Collins has since then demonstrated the reliability of this model in several publications.^7^ Others have recently published similar data on primary prostate cells, using the basal cell marker CD49f. In contrast to Smith and colleagues,^8^ our data showed that all subpopulations isolated from our primary cells expressed the same levels of CD49f, in agreement with Taylor *et al.*^7, 9^ and Collins *et al.*^12^ who also demonstrated CD49f expression on all the cell subpopulations previously described by Collins and Maitland.^12, 13, 28^ This implies that, despite the use of different basal cell markers, both groups are ultimately describing the same populations of prostate epithelial cells.

Cytokines play an important role in the development of therapy resistance.^52, 53^ Current therapies such as radiation and docetaxel can trigger an inflammatory response, and an increase in cytokine levels of IL-6, IL-8 and IL-4 have all been reported.^15, 20, 29, 54, 55^ *In vitro* studies demonstrated that, in cell lines, IL-4 enhances the DNA repair activity triggered by radiation therapy.^56^ In this study, we observed that IL-4 had no influence on clonogenic recovery of primary PCa cells following irradiation. Similarly, IL-4 also demonstrated no influence on the clonogenic recovery potential of primary PCa cells following docetaxel treatment. We therefore conclude that IL-4 does not have a direct influence on therapy resistance in PCa.

Here we demonstrate for the first time that activation of STAT6 by IL-4 from the local microenvironment results in a significant increase in the colony-forming ability of primary PCa cells. This finding describes a novel role of the IL-4/IL4Rα/STAT6 axis in the highly tumourigenic progenitor population of PCa. However, the specific role of STAT6 in rare CSCs has to be clarified in further studies, in order to investigate STAT6 as a potential novel therapeutic target. We have also further demonstrated the complexity of PCa tumour initiation and progression, which highlights the importance of targeting not only the epithelial cell populations but also the tumour microenvironment.

## Materials and methods

### Culture of cell lines and primary prostate cells

The PCa cell lines LNCaP, PC3 and Du145 were obtained from the American Type Culture Collection (Rockville, MD, USA), cultured, and authenticated as previously described.^57^ The LNCaP sub cell line LNCaP IL-6^+^ was derived after long-term treatment with IL-6.^58^ 293FT cells were cultured as described by Invitrogen (Life Technologies Ltd, Paisley, UK).

Benign and cancerous primary prostate cells were cultured as described previously.^6, 13, 28^ Tissues were obtained with patients' consent and full ethical approval (Yorkshire and Humber NHS Research Ethics Office—NRES number 07/H1304/121) from patients. Stem cell populations (SC, CD49b^high^/CD133^+^), transit-amplifying populations (TA, CD49b^high^/CD133^−^), and committed basal populations (CB, CD44^+^/CD49b^low^/CD133^−^) were obtained via cell fractionation using the protocol previously published by Richardson *et al.*^28^ Patient donor details are listed in Supplementary Table 1.

### Live cell count

Collected cells were stained with Trypan Blue (Sigma-Aldrich Company Ltd, Gillingham, UK) and counted using a Neubauer’s haemocytometer. Unstained cells were seeded for subsequent experiments.

### Irradiation of cells

Cells were irradiated using an RS2000 X-Ray Biological Irradiator, containing a Comet MXR-165 X-Ray Source (Rad-Source Technologies Inc., Suwanee, GA, USA). A dose of 2.5, 5, 10, or 60 Gy was administered with a dose rate of 0.02 or 0.08 Gy/ s.

### STAT6 inhibitor and docetaxel treatment of cells

STAT6 inhibitor (AS1517499, Axon Medchem, Groningen, the Netherlands) and docetaxel (Sigma-Aldrich Company Ltd) were dissolved in dimethyl sulfoxide (DMSO, Sigma-Aldrich Company Ltd). For the STAT6 inhibitor experiments, doses of 10, 30, 100 and 300 nm were used. For docetaxel experiments, doses of 1.25, 2.5 , 5 , 10 , 100 and 1250 nm were used.

### Cloning strategy of pGLTR-IL-4-PURO and pGLTR-GUS-PURO

For cloning of human IL-4 complimentary DNA into the lentiviral vector pGLTR-X-PURO, the Invitrogen Gateway Recombination Cloning Technology (Life Technologies Ltd) was used. pGLTR-X-PURO was kindly provided by Dr Stephan Geley.^59^ IL-4 was re-cloned from pcD-human-IL-4(clone 125) (ATCC, Rockville, MD, USA) using a pair of specific primers (5′-CAAAAAAGCAGGCTCCATGGGTCTCACCTCCCAAC-3′ and 5′-CAAGAAAGCTGGGTCTCAGCTCGAACACTTTGAATATTTC-3′), which incorporated specific overhangs for the gateway reaction into the pDONOR221. The pDONOR221-*Escherichia* *coli*-β-glucuronidase (GUS) was used for the control vector pGLTR-GUS-PURO and was provided by Invitrogen (Life Technologies Ltd).

### Lentivirus production and generation of stable cell lines

For production of lentiviral particles, 293FT cells were transfected with psPAX2, pVSV-G and the corresponding pGLTR-X-PURO expression vector using the calcium phosphate transfection method.^60^ psPAX2 was a gift from Didier Trono (Addgene plasmid # 12260) and pVSV-G was a gift from Robert Weinberg (Addgene plasmid # 8454).^61^ Viral supernatants were harvested 48 h after transfection and passed through a 0.45 μm filter. STO cells were incubated for 8 h with the virus in the presence of 2 μg/ml polybrene and subsequently selected with 5 μg/ml puromycin (Sigma-Aldrich Company Ltd).

### [^3^H] Thymidine incorporation assay and MTT Assay

PC3, Du145 and LNCaP IL-6^+^ cell lines were seeded at a density of 2 × 10^3^ cells per well and LNCaP cells at 10 × 10^3^ cells per well in triplicate onto 96-well plates. On the following day, cells were treated with different concentrations of IL-4 (0, 0.1, 1; 5, 10 ng/ml, Sigma-Aldrich Company Ltd) and were incubated for 96 h.

To measure DNA synthesis, [^3^H] labelled thymidine (1 μCi per well) was added to the wells 12 h before harvesting. The DNA was harvested on UniFilter 96-well filter plates (Perkin-Elmer, Vienna, Austria) and 50 μl Scintillation fluid was added. The radioactivity was quantified using a Chameleon 5025 liquid scintillation counter (HVD Life Sciences, Vienna, Austria).

For the MTT Assay the EZ4U Proliferation Assay (Biomedica, Vienna, Austria) was used and performed following the manufacturer’s protocol.

### Western blot analysis

For Western blot analysis cells were washed with Phosphate-buffered saline and lysed in Radioimmunoprecipitation assay buffer supplemented with complete Mini EDTA-free protease inhibitor tablets (Roche, Welwyn Garden City, UK) and the phosphatase inhibitor cocktail PhosSTOP (Roche). Protein quantification and Western blot was performed as described earlier.^62^ Antibodies used are listed in Supplementary Table 2. For the Western blot experiments STO-free cultures were used and treated with 48 h-conditioned media from STO-GUS or STO-IL-4.

### Flow cytometry

Flow cytometry for IL4Rα (CD124) was performed as described before by Kroon *et al.*^18^ Antibodies used are listed in Supplementary Table 2. All experiments were performed in at least three independent biological replicates unless otherwise specified.

### Clonogenic and clonogenic recovery assays

The clonogenic potential and clonogenic recovery assays were performed as previously described.^63^ About 200 cells were plated from semi-confluent primary prostate cells onto 6-well collagen I-coated plates (BD Biocoat; BD Biosciences, San Jose, CA, USA) in the presence of irradiated STO cells. Colonies were counted after 15 days and recorded if they contained more than 32 cells (five population doublings).

### Cell migration and invasion assays

Cell migration and invasion assays were performed as previously described.^64^ All experiments were performed in at least three independent biological replicates unless otherwise specified.

### Quantitative real-time PCR for mRNA

Total RNA was extracted from cells using Qiagen RNeasy Mini Kit (Qiagen, Manchester, UK) according to the manufacturer's protocol. RNA was reverse transcribed using random hexamers (Life Technologies Ltd) and the Reverse Transcriptase Kit SuperScript III (Life Technologies Ltd).

Quantitative real-time PCR was conducted using TaqMan gene expression assays (Life Technologies Ltd) and the iTaq Universal Supermixes (Bio-Rad Laboratories Ltd, Hertfordshire, UK), according to the manufacturer's protocol.

### Proteome profiler human Phospho-Kinase Array

For the Proteome Profiler Human Phospho-Kinase Array (R&D, Abingdon, UK) 1 × 10^6^ primary PCa cells were seeded in a collagen I-coated 10 cm-well plate and treated with 48 h-conditioned media from STO-IL-4 or STO-GUS for 30 min or 48 h. The array was performed according to the manufacturer’s manual and developed in a GeneGnome XRQ imaging system (Syngene, Cambridge, UK).

### Tissue microarray and immunohistochemistry

The construction of the TMA for IL4Rα was performed as previously described.^57^ Georg Schäfer (Institute of Pathology, Medical University Innsbruck, Austria) evaluated the TMA by using the semiquantitative scoring system ‘quickscore’ by multiplication of the proportion of positive cells and the average staining intensity.^65^

### Statistical analysis

Prism 6 (GraphPad Software, La Jolla, CA, USA) was used for statistical analyses and the evaluation of the IC50. Gaussian distribution was determined using Kolmogorov–Smirnov test. Mann–Whitney *U* or Student’s *t*-test (two-sided) were used to determine whether two sets of data were significantly different from each other. For correlation analysis (Pearson’s method) SPSS (IBM United Kingdom Ltd, Hampshire, UK) was used. Data are presented as mean±s.d. or mean±s.e.m. unless otherwise specified. Mean±s.d. was used to describe the distribution of the sample values in an experiment and s.e.m was used to estimate how variable the means were in multiple repeated experiments.^66^ *P*-values of <0.05 were considered significant. All differences highlighted by asterisks were statistically significant as encoded in figure legends (*P<0.05; **P<0.01; ***P<0.001). All experiments were performed in at least three independent biological replicates unless otherwise specified.

## Figures and Tables

**Table 1:**
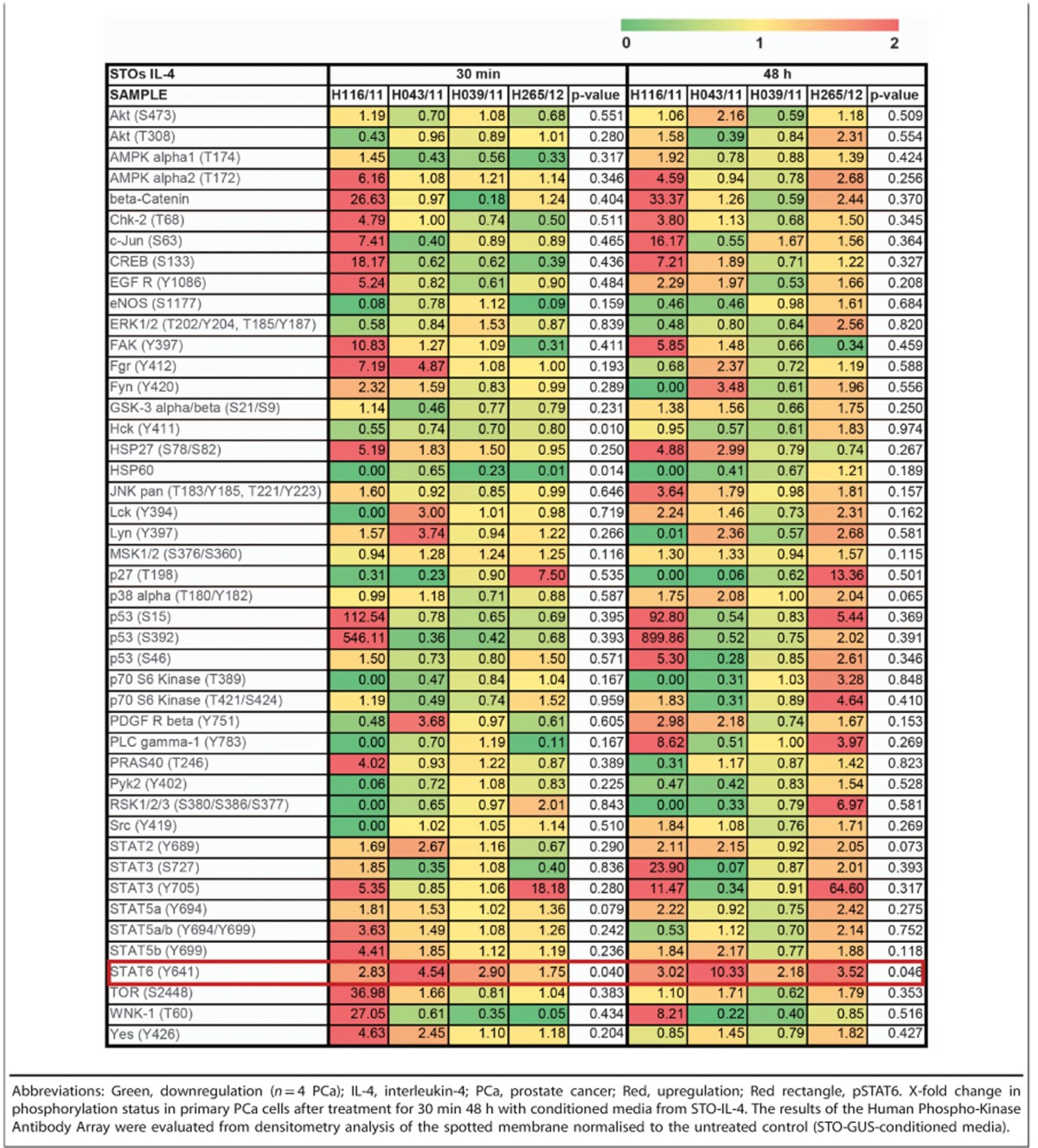
Evaluation of the Human Phospho-Kinase Antibody Array after treatment of primary PCa cells with IL-4
