# Supplementary figures and images for: The immunosuppressive cytokine interleukin-4 increases the clonogenic potential of prostate stem-like cells by activation of STAT6 signalling
Source: Oncogenesis. 2017 May 29;6(5):e342–. doi: 10.1038/oncsis.2017.23 (PMC5523058; doi:10.1038/oncsis.2017.23)

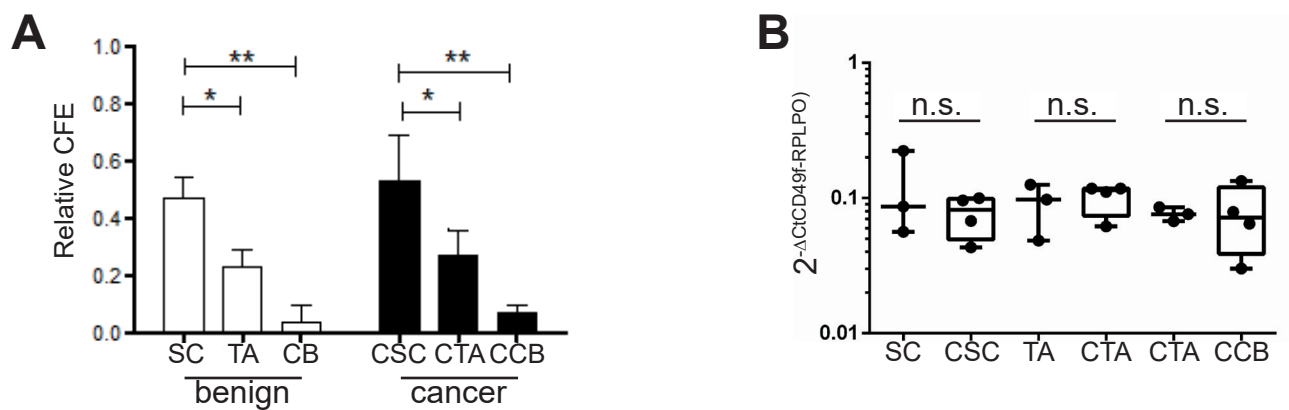

(n.s.: not significant; \*:  $p < 0.05$ ; \*\*:  $p < 0.01$ ; \*\*\*:  $p < 0.001$ )

Supplement: Supplementary Figure 1 [file oncsis201723x1.pdf]

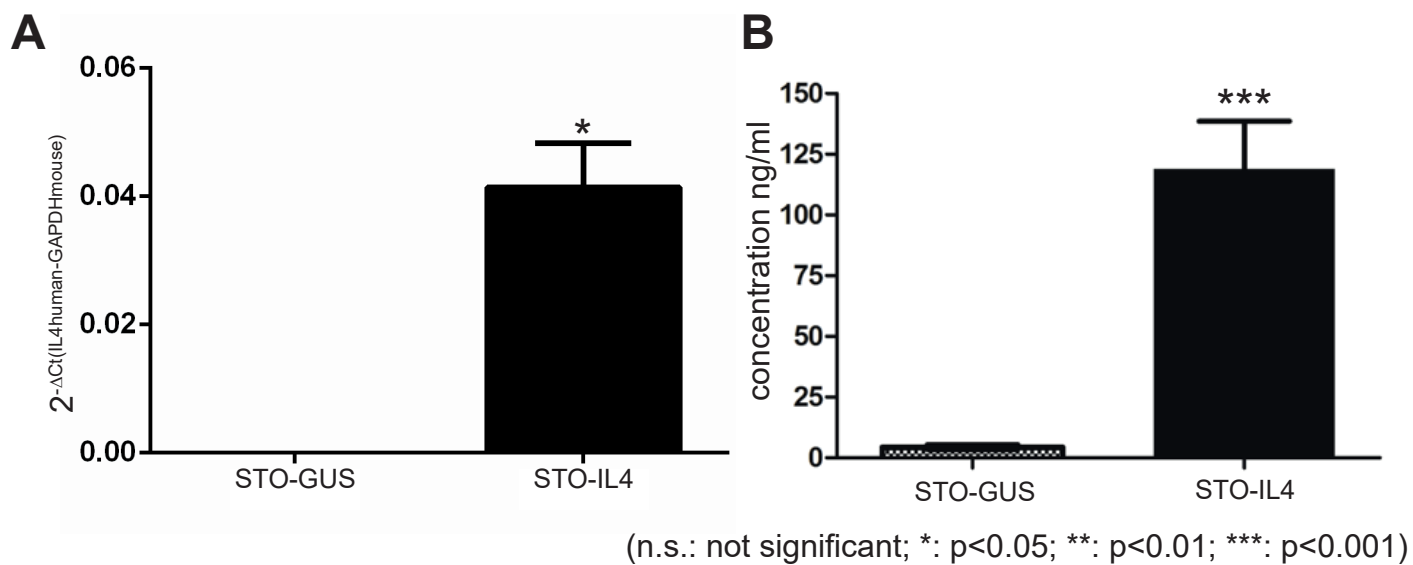

Supplement: Supplementary Figure 2 [file oncsis201723x2.pdf]

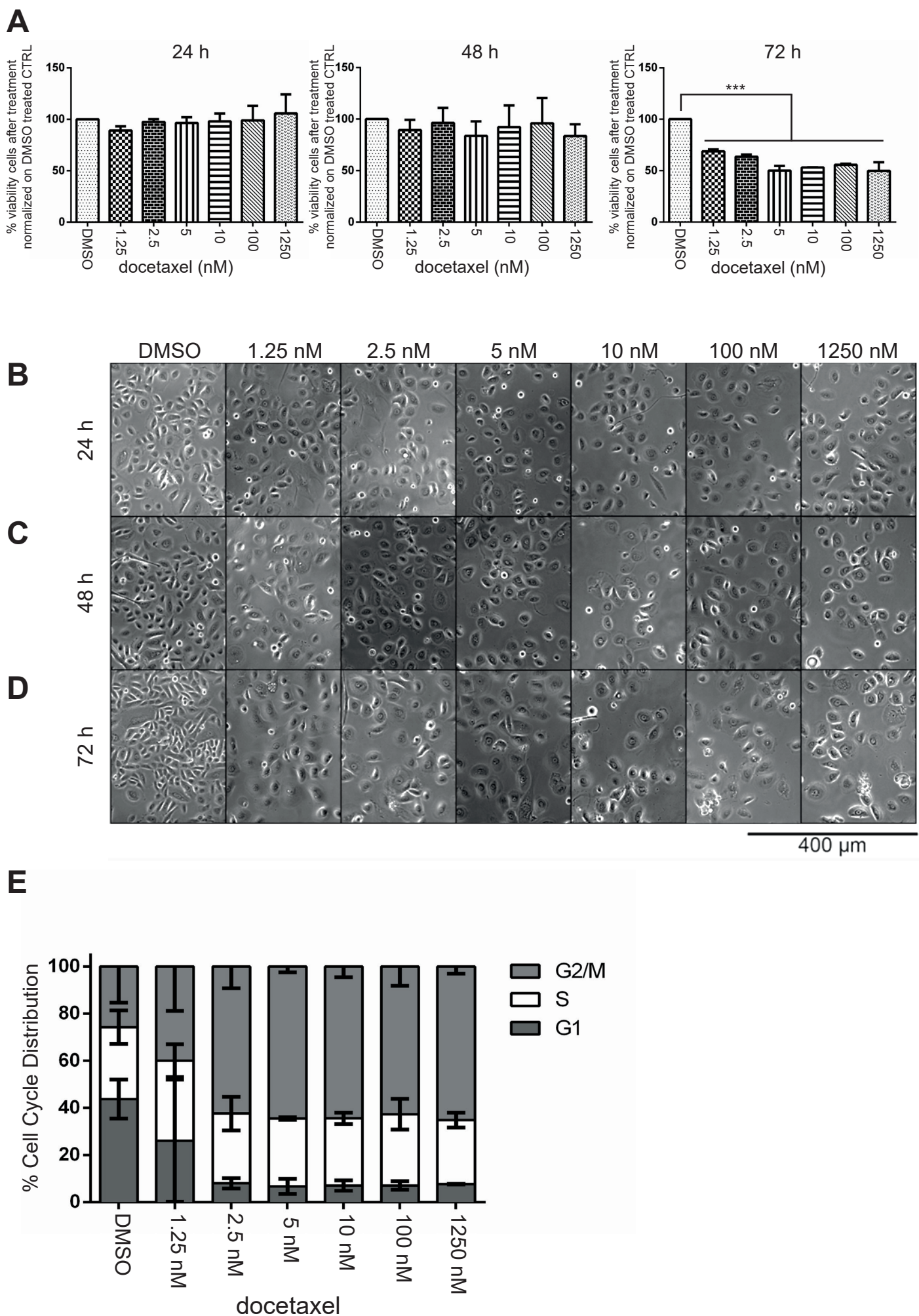

(n.s.: not significant; \*:  $p < 0.05$ ; \*\*:  $p < 0.01$ ; \*\*\*:  $p < 0.001$ )

Supplement: Supplementary Figure 5 [file oncsis201723x5.pdf]

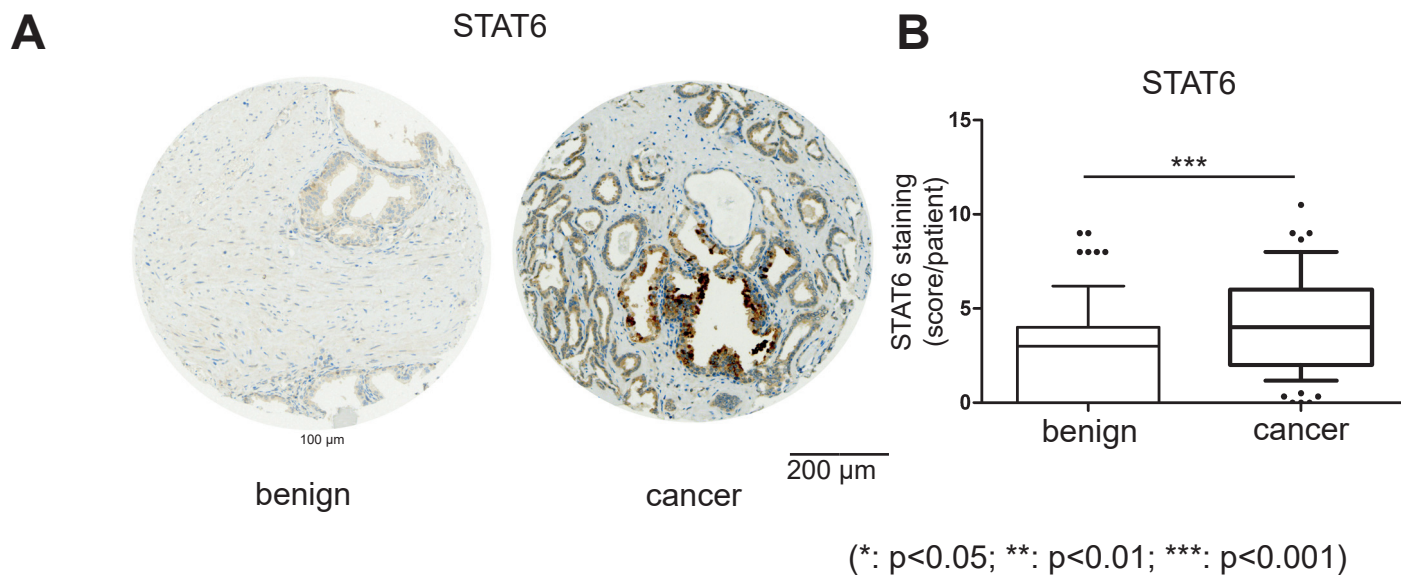

Supplement: Supplementary Figure 6 [file oncsis201723x6.pdf]

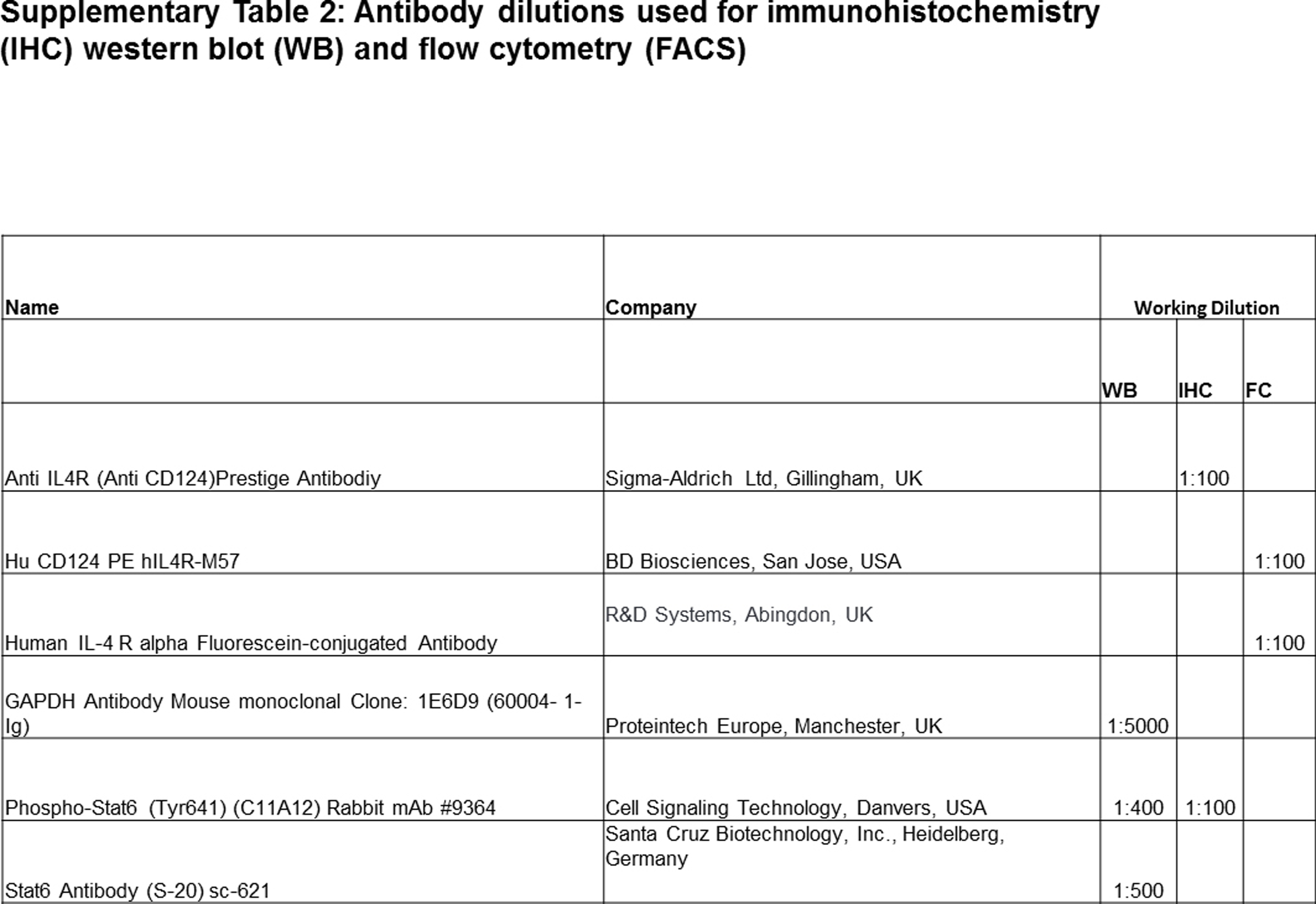

Supplement: Supplementary Table 2 [file oncsis201723x8.tif]
