# Supplementary material for: The immunosuppressive cytokine interleukin-4 increases the clonogenic potential of prostate stem-like cells by activation of STAT6 signalling
Source: Oncogenesis. 2017 May 29;6(5):e342–. doi: 10.1038/oncsis.2017.23 (PMC5523058; doi:10.1038/oncsis.2017.23)
Supplement: Supplementary Figure 3 [file oncsis201723x3.pdf]

Supplementary Figure 3: IL-4 influences clonogenic potential but not proliferation

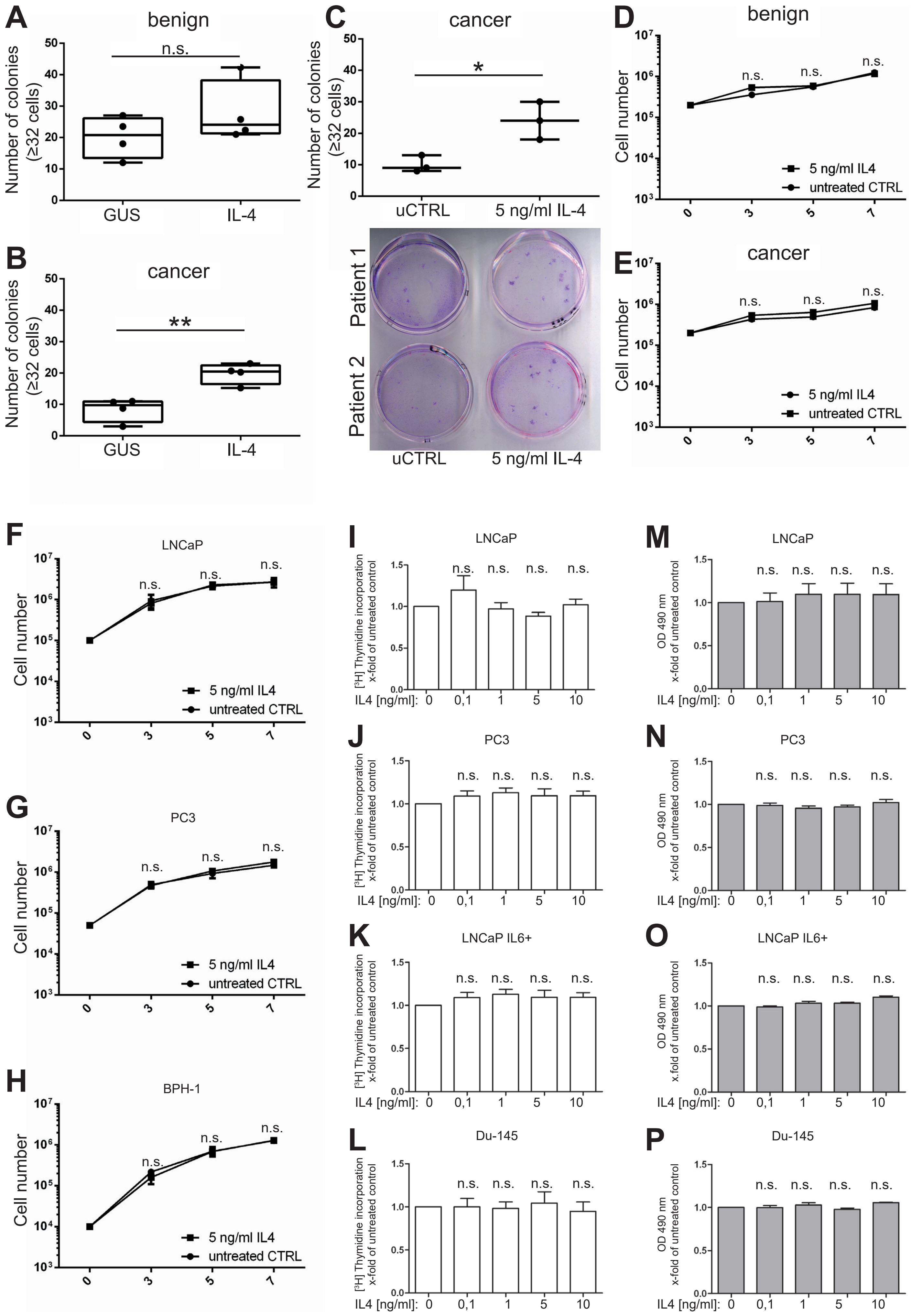

(n.s.: not significant; *: p<0.05; **: p<0.01; ***: p<0.001)
