# Supplementary material for: The immunosuppressive cytokine interleukin-4 increases the clonogenic potential of prostate stem-like cells by activation of STAT6 signalling
Source: Oncogenesis. 2017 May 29;6(5):e342–. doi: 10.1038/oncsis.2017.23 (PMC5523058; doi:10.1038/oncsis.2017.23)
Supplement: Supplementary Figure 4 [file oncsis201723x4.pdf]

Supplementary Figure 4: Human IL-4 levels in STO-IL4 diluted with STO-GUS

A

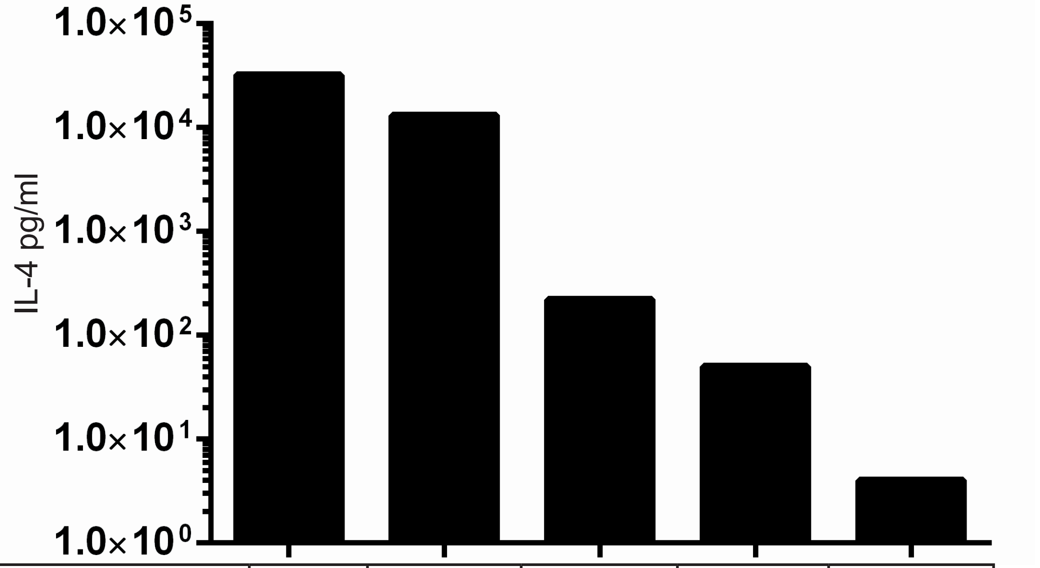

Dilution Factor	[STO-GUS:STO-IL4]:	$1{:}10^0$	$1{:}10^1$	$1{:}10^2$	$1{:}10^3$	$1{:}10^4$
IL-4 Concentration	[ng/ml]:	32.00	13.00	2.2	0.5	0.04

B

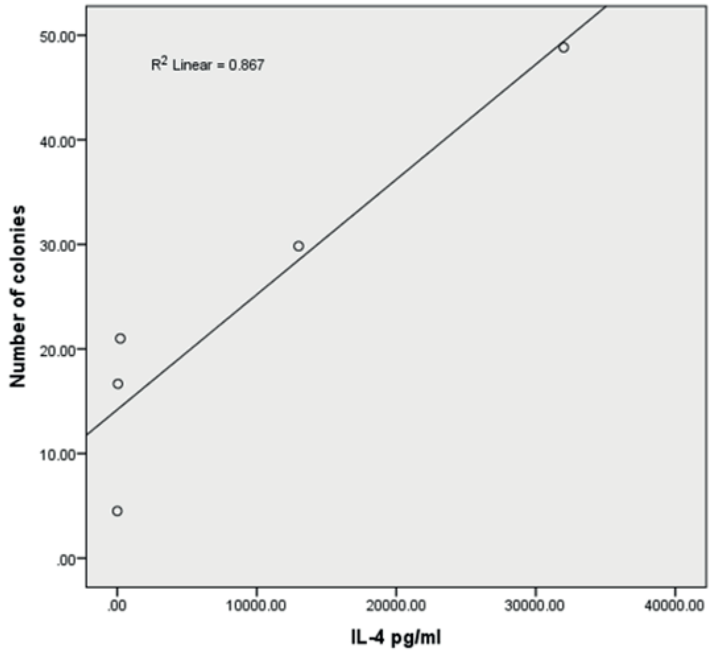

C

Correlations

		Colonies	Concentration
Colonies	Pearson Correlation	1	.931*
	Sig. (2-tailed)		0.021
	N	5	5
Concentration	Pearson Correlation	.931*	1
	Sig. (2-tailed)	0.021	
	N	5	5

*. Correlation is significant at the 0.05 level (2-tailed).
