# Supplementary material for: The immunosuppressive cytokine interleukin-4 increases the clonogenic potential of prostate stem-like cells by activation of STAT6 signalling
Source: Oncogenesis. 2017 May 29;6(5):e342–. doi: 10.1038/oncsis.2017.23 (PMC5523058; doi:10.1038/oncsis.2017.23)
Supplement: Supplementary Table 1 [file oncsis201723x7.pdf]

Supplementary Table 1: Patients

Name	BPH	Cancer	GS	Passage No	Clonogenic Assays	Migration and Invasion Assays	Clonogenic Recovery Assay	Phospho Array	qRTPCR
559015	✓			0	✓				
423011	✓			0	✓				✓
14009	✓			0	✓				✓
226011	✓			0					✓
114011	✓			0					✓
215012	✓			0					✓
53011	✓			0					✓
62011	✓			0	✓				✓
087011 LB		✓	3+4	0	✓				✓
568015 RM		✓	3+4	0	✓				
567015 RM		✓	4+4	0	✓				✓
045011 LB		✓	3+4	0	✓				
023010 RM		✓	5+4	0	✓				✓
262012 RA		✓	3+4	0	✓				
39011 LB		✓	4+5	0	✓			✓	
239012 RA		✓	4+4	0	✓				
134011 LM		✓	4+3	0	✓				
066011 RB		✓	4+3	0	✓				✓
043011 RB		✓	4+4	0	✓			✓	
265012 RA		✓	3+4	0	✓			✓	
116011 LB		✓	4+3	0	✓			✓	
06811 RB		✓	4+3	0	✓				✓
110011 RA		✓	4+3	0	✓		✓		
045011 RB		✓	4+3	0	✓		✓		✓
048011 RA		✓	3+3	0	✓		✓		
087011 LB		✓	4+3	0	✓		✓		
0422014 LM		✓	4+3	0			✓		
254012 LM		✓	3+3	0			✓		
070011 RA		✓	3+4	0		✓			
137011 LM		✓	4+3	0		✓			
063011 RB		✓	3+3	0		✓			
103011RA		✓	3+5	0		✓			
116011 RA		✓	4+3						✓
219012 LB		✓	4+3						✓
041011 LA		✓	3+4						✓
